# Efficacy of Birth Dose Vaccination in Preventing Mother-to-Child Transmission of Hepatitis B: A Randomized Controlled Trial Comparing Engerix-B and Sci-B-Vac

**DOI:** 10.3390/vaccines9040331

**Published:** 2021-04-01

**Authors:** Rifaat Safadi, Tawfik Khoury, Nizar Saed, Marwan Hakim, Jeryes Jamalia, Yousef Nijim, Nicola Farah, Tawfik Nuser, Nidaa Natur, Mahmud Mahamid, Johnny Amer, Pia L. Roppert, Wolfram H. Gerlich, Dieter Glebe

**Affiliations:** 1 Liver Unit, Institute of Gastroenterology, Hadassah-Hebrew University Hospital, Jerusalem 91120, Israel; safadi@hadassah.org.il (R.S.); nidaa@hadassah.org.il (N.N.); joni@hadassah.org.il (J.A.); 2 Liver Unit, Holy Family Hospital, Nazareth 1613101, Israel; dr.nizarsaad@yahoo.com (N.S.); n.farah@hfhosp.org (N.F.); mmahamid@szmc.org.il (M.M.); 3 Galilee Medical Center, Department of Gastroenterology, Nahariya 22100, Israel; 4 Faculty of Medicine in the Galilee, Bar-Ilan University, Safed 1311502, Israel; 5 Nazareth Hospital, Nazareth 1613101, Israel; mhakim@bezeqint.net (M.H.); yousifnijim@nazhosp.com (Y.N.); 6 French Hospital, Nazareth 1613101, Israel; jjeryes@st-vincent-hospital.com (J.J.); nseirt@yahoo.com (T.N.); 7 The Cheryl Spencer Department of Nursing, Faculty of Social Welfare and Health Science, University of Haifa, Haifa 3498838, Israel; 8 Shaare Zedek Medical Center, Department if Gastroenterology and Liver Diseases, Faculty of Medicine, Hebrew University of Jerusalem, Jerusalem 91120, Israel; 9 Dajani Hospital, Jerusalem 91120, Israel; 10 National Reference Center for Hepatitis B Viruses and Hepatitis D Viruses, Institute of Medical Virology, Justus Liebig University Giessen, 35392 Giessen, Germany; p_roppert@gmx.de (P.L.R.); wolfram.h.gerlich@viro.med.uni-giessen.de (W.H.G.); dieter.glebe@viro.med.uni-giessen.de (D.G.); 11 German Center for Infection Research (DZIF), Partner Site Giessen-Marburg-Langen, 35392 Giessen, Germany

**Keywords:** HBV, vaccines, transmission, vertical, infection

## Abstract

*Background and aims:* Peripartum transmission of hepatitis B virus (HBV) from an infected mother to the child can be prevented in most but not all cases by immediate vaccination of the newborn. The aim of this study was to compare the efficacy of two licensed hepatitis B vaccines, Engerix-B versus Sci-B-Vac, in preventing peripartum HBV transmission. *Methods:* A prospective multicenter randomized controlled study in 4 delivery centers was performed from 2009 to 2014. HBsAg positive pregnant women and their newborns were recruited at the delivery rooms. All newborns received Hepatitis B Immune Globulin within 10 h after birth, as well as active HBV vaccination at 0, 1 and 6 months of age. Maternal assessment at delivery included transaminases, blood count, international normalized ratio and viral status. Infants were tested for HBsAg, anti-HBc and anti-HBs at 12 months of age. *Results:* In the intention to treat (ITT), 171 infant and mother pairs fulfilled the study enrollment criteria and completed follow up, 82 received Engerix-B and 89 Sci-B-Vac. Maternal parameters and viral status were similar in both groups. At 12 months of age, the Sci-B-Vac group had lower HBsAg carriage rates (1/89, 1.1%) than the Engerix-B group (5/82, 6.1%) with borderline significance (risk difference of −0.05, 95% CI −0.11–0.007, *t*-test = 0.05), and borderline significance lower vaccine failure rates with anti-HBs < 10 mIU/mL in the Sci-B-Vac (2/89, 2.2%) than in the Engerix-B (8/82, 9.8%, *p* = 0.05). Higher seroprotection rates were found in the Sci-B-Vac group with all anti-HBs titer stratifications of >10 mIU/mL (*p* = 0.05), >100 mIU/mL (*p* = 0.05) and >1000 mIU/mL (*p* = 0.01). Active/passive vaccination was effective in 10/13 cases with maternal HBV DNA levels > 7 log10 IU/mL up to 9.5 log10 IU/mL, but failed in 3 cases for unknown reasons. *Conclusion:* Sci-B-Vac was superior to Engerix-B in preventing peripartum HBV transmission in neonates from HBsAg+ mothers and induces significantly higher anti-HBs levels. NIH registration number: NCT 01133184.

## 1. Introduction

The World Health Organization’s (WHO) 2017 hepatitis report indicates that 257 million people are chronically infected worldwide with the hepatitis B virus (HBV) [1]. Mother to child HBV transmission (MTCT) in early childhood is the leading transmission route in many high prevalence regions like East Asia and leads to a high rate of chronic HBV infections [2,3]. Immigrations from Asia to developed countries significantly increased HBV prevalence in the United States and Europe [4,5]. Since 1992, many countries adapted universal vaccination by using active HBV vaccines in all newborns within 24 h of birth, followed by at least two additional doses at ages of 1 and 6 months [6]. In addition, passive immunization with hepatitis B immunoglobulin is given within 12 h after birth in case of high-risk neonates born to hepatitis B surface antigen (HBsAg) positive mothers [7]. The MTCT risk was reduced from 90% to 5–10% following universal vaccination. However, it remains a concern for 8–15% of infants in high-risk groups who are born to highly viremic mothers, for instance, women who are positive for hepatitis B e antigen (HBeAg+) and/or have high HBV DNA levels [7]. Therefore, further efforts in preventing MTCT may have significant implications in the global control of HBV. In Israel, MTCT was estimated to occur in 5% of HBsAg positive mothers despite the use of vaccination and antiviral HBV therapy in highly viremic pregnant women [8]. The current standard 2nd generation active hepatitis B vaccines contain the small HBsAg protein and are produced in genetically engineered yeast cells [9], with Engerix-B being one of the standard of care vaccines. Sci-B-Vac (previously known as Bio-Hep-B [10]) is a 3-antigen HBV vaccine that is produced in a mammalian cell line and contains all three HBV surface proteins, including the preS1, preS2 domains as well as the small HBsAg. Based on superior immunogenicity in neonates [9,10], Sci-B-Vac was introduced in Israel in 2010 as an alternative to Engerix-B [11] in neonatal vaccination.

Our study’s goal was to compare the efficacy of these two active hepatitis B vaccines in preventing MTCT in neonates born to HBV carrier mothers.

## 2. Materials and Methods

This study was a prospective, randomized, multicenter, open label, parallel group study. Paired participants (HBsAg+ mothers and newborns) were recruited at the delivery rooms of four Israeli hospitals between 2009 and 2014; Dajani Hospital in Jerusalem, Holy Family Hospital in Nazareth, Nazareth Hospital and French Hospital in Nazareth participated in the study. Confirmed HBsAg positive pregnant women (tested by community laboratories) and their newborns were recruited at delivery rooms.

The mothers and the newborns were randomly assigned (1:1) into one of two treatment groups using computer based random allocation sequence. The allocation was concealed until the interventions were assigned. The participants were enrolled by the treating physician at each center, while a study coordinator at each center which was blinded to the participants enrolled, randomly assigned participants to either group. After randomizing and assigning treatment, both treating physicians and the participants were unblinded to the type of vaccine administered. All mothers had HBV DNA levels tested at the delivery room. All mothers who were followed by an outpatient hepatologist and who had >7 log10 IU/mL were offered treatment with anti-viral therapy starting at mid-pregnancy around week 20 until 6 months after delivery.

**Figure 1:**
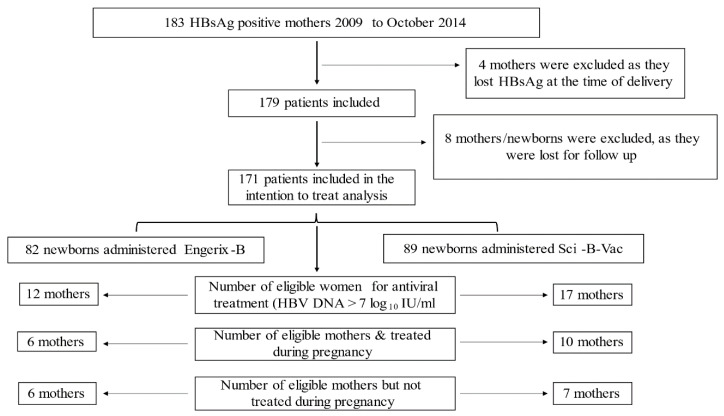
The intention to treat analysis study flowchart.

Group A newborns received three doses intramuscular (IM) of Engerix-B (10 µg/0.5 mL) within 12 h after delivery, and at ages one and 6 months; group B newborns received three IM doses of Sci-B-Vac (5 µg/0.5 mL) at the same intervals. Cases that by a mistake received different vaccines were excluded, see Figure 1. In addition, 200 IU hepatitis B immune globulin (HBIG, BayHep B) were administered IM to all newborns within 24 h after birth. Shortly before delivery, all mothers were tested for HBsAg, HBeAg, antibody to hepatitis B e antigen (anti-HBe), antibody to hepatitis C virus (anti-HCV) and antibodies to hepatitis D virus (anti-HDV) using the chemiluminescent microparticle immunoassay with Architect Reagent Kits. Additionally, pre-delivery maternal HBV DNA levels were assessed by Abbott Real-Time HBV Viral Load Amplification Reagent Kit, Abbott, with lower limit of quantitation 15 IU/mL). Moreover, maternal complete blood count, international normalized ratio (INR), alanine aminotransferase (ALT) and aspartate transferase (AST) were performed prior to delivery. Newborns were followed for up to 1 year post-delivery for vaccine response by evaluating HBsAg, antibody to HBsAg (anti-HBs) levels and hepatitis B core antibody (anti-HBc) using chemiluminescent microparticle immunoassay 254 with Architect Reagent Kits, Abbott. HBsAg values < 0.05 international units/mL were considered negative, while values ≥ 0.05 were considered positive.

Serum samples were analyzed for a 500-nucleotide long sequence of the virus polymerase and surface protein nucleotide sequence by polymerase chain reaction (PCR) as described [12]. This genomic region represents the reverse transcriptase domain of HBV and the HBs-antigenic loop. In brief, HBV DNA from serum samples was purified with the High Pure Viral Nucleic Acid Kit (Roche, Mannheim, Germany), following the manufacturer’s operating manual. Amplification of the indicated HBV genome sequence was done using Phusion High-Fidelity DNA polymerase (Thermo Scientific, Langenselbold, Germany). Sequencing of HBV PCR products was done by LGC Genomics (Berlin, Germany). Resulting HBV sequences were analyzed with Lasergene 10 (DNASTAR, Madison, WI, USA).

The study protocol conformed to the ethical guidelines of the 1975 Declaration of Helsinki and was approved by the local Ethics Committee of each institute (Trial registration number: 0509-08-HMO). Informed consent was waived by the local ethical committee given that the vaccines that were administered at each center was a standard of care practice. All authors had access to the study data and had reviewed and approved the final manuscript.

### 2.1. Participant Population

Inclusion criteria included pregnant women who had a chronic HBV infection with HBsAg positivity documented by community laboratories during the first trimester of pregnancy and confirmed in the delivery room (tested in the Hadassah Medical Organization laboratory, Jerusalem), and adhered to the trial protocol, were eligible to participate in the study with newborns. Exclusion criteria included women with other hepatic diseases including, alcoholic liver disease, drug-induced liver injury, autoimmune hepatitis, non-HBV viral hepatitis, cholestatic liver diseases and metabolic/genetic liver disease. Furthermore, we excluded mothers with any other immunity-related disease and those treated with immune suppression. The metabolic background was similar in both groups, including hypertension, diabetes and hyperlipidemia. The delivery room attendance was the first study event for recruitment, screening and randomization timepoints. The previous HBV follow up during pregnancy was variable in the community and not within the study protocol.

### 2.2. Primary Endpoint

The primary endpoint was the rate of HBV infections in the infants after 12 months following active vaccination as per the described regimen in the intention to treat analysis (ITT). The rate of HBV infections was defined as the proportion of infants who had detectable serum HBsAg at 12 months of age using the chemiluminescent microparticle immunoassay with Architect Reagent Kits, Abbott.

### 2.3. Secondary Endpoint

To assess the rates of positive anti-HBc and the vaccine seroprotection rate defined as anti-HBs titer > 10 IU/mL at 12 months of age in the ITT analysis. Moreover, we also aimed to assess possible factors related to the outcome.

### 2.4. Statistical Analysis

All enrolled newborns were included in the intention to treat analysis of the rates of mother-to-child transmission, as well pre protocol analysis (PPA) was reported. Quantitative variables are expressed as median and range, and categorical variables are expressed in percentages. Categorical variables were compared by applying the Fisher exact test. *p-*values of 0.05 or less were considered statistically significant. All *p-*values and confidence intervals were based on two-tailed tests. Wilcoxon rank-sum was used to compare the median values. Regarding sample size calculation, taking into account that almost 87% seroprotection rate of Engerix-B vaccine from real-life data has been demonstrated, we calculated that 87 subjects in each group (total of 174 subjects) are required to have a 80% chance of detecting, as significant at the 5% level, an increase in the primary outcome measure from 87% response rate in the Engerix-B group to 98% in the Sci-B-Vac group.

## 3. Results

### 3.1. Participants

In the ITT analysis, altogether 183 HBsAg positive pregnant women were identified by community laboratories within the first trimester of pregnancy and were tested again for HBsAg at the time of delivery at the Hadassah Medical Organization laboratory, Jerusalem. Four mothers (2.2%) were excluded, because they had lost HBsAg at the time of delivery. An additional 16 women/newborns were excluded from the study, as 8 babies received two different active vaccines by mistake of whom we have their data regarding HBV status at one year and 8 were lost for follow up early in the study and from whom we lack data. Therefore, in the ITT, a total of 171 women were included in the analysis whereas only 163 mother-infant pairs were included in the per protocol analysis (PPA) because to 8 infants two different vaccines were administered. In the ITT, Group A included 82 newborns who were allocated to the Engerix-B vaccine and all of these infants received the Engerix-B. Group B included 89 newborns, who were allocated to the Sci-B-Vac, of whom 81 received the Sci-B-Vac and 8 received the Engerix-B (Figure 1). There were no HBV vaccines related adverse events encountered throughout the study.

### 3.2. Maternal Characteristics

In the ITT, the median ± interquartile range (IQR) age on delivery in group A was 27.03 ± 9.53 years compared to 28 ± 8.02 years in group B (*p* = 0.15). The levels of hemoglobin, platelet count, leukocyte count and liver enzymes were similar between the two groups in the ITT and PPA analysis (*p* = NS), while the INR level were slightly lower in group B (*p* = 0.02 in the ITT and 0.03 in the PPA analysis, respectively, Table 1). Although those values appear in the statistical evaluation as significant differences, they are within the normal limit and unlikely to have an impact in the results of this study.

**Figure 2:**
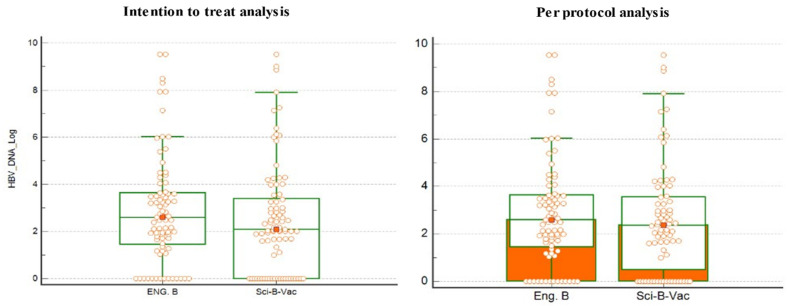
The median and interquartile range (IQR) of maternal hepatitis B virus (HBV) DNA viral load measurement at delivery. There was no significant difference in the log10 median. Eng. B, Engerix-B. Wilcoxon rank-sum was used to compare the median values.

In the ITT analysis, the HBeAg positivity rate indicating high HBV infectivity was slightly higher in group B (10/89, 11.24%) vs. (7/81, 8.54%), whereas there was no statistical difference in the anti-HBe positivity rates (90.24% vs. 77.53%, *p* = 0.58) in groups A and B, respectively. The median level of HBV viral loads was also similar in the two groups (Table 1). The median of HBV DNA levels was not different in both groups (2.6 in group A vs. 2.1 log 10 IU/mL in group B, *p* = 0.19). In the PPA, the results were almost similar to the ITT analysis (Table 1). Figure 2 demonstrate the median ± IQR of maternal HBV viral load in the ITT analysis. Per log_10_ measurements at delivery were also similar between cohorts. HBV DNA > 5 log_10_ IU/mL as marker of potential MTCT was found in 12/82 (14.6%) vs. 11/89 (12.4%) of mothers in group A and group B, respectively in the ITT analysis, and 12/82 (14.6%) vs. 10/81 (12.3%) in groups A and B, respectively, in the PPA. On the other hand, 84.1% in group A in the ITT and PPA and 83.1% and 82.7% in the ITT and PPA respectively, had HBV DNA < 5 log_10_ IU/mL. Of note, 13 women had >7 log_10_ IU/mL at delivery. They did not receive anti-viral treatment as they had not been monitored by a hepatologist or refused therapy. Sixteen women were treated with nucleoside analogues (NUCs) due to high viremia >7 HBV DNA log_10_ at weeks 20 of pregnancy. Anti-viral treatment was discontinued 6 months after delivery. Pre-delivery treatment with lamivudine and telbivudine was administered at equal frequencies in groups A and B (2 and 4 patients, respectively) and 4 patients were administered tenofovir in the Sci-B-Vac group.

Genotype D and subgenotype D1 of HBV is predominant in Israel [13]. This is true for the current study population group as well. A selective analysis in nine cases (Table 2) confirmed genotype D in all cases, 77.8% (7/9) had subgenotype D1 and the rest D2. The HBsAg subtype ayw2 was found in 88.9% (8/9) and one case with ayw3. Mutations within the reverse transcriptase domain of HBV and the HBs-antigenic loop are illustrated in Table 2. Maternal and infant characteristics of the mothers who had genetic testing performed are shown in Table 2 as well. Those mutations are not known as vaccine immune escape mutations, but one HBsAg mutation was unusual because T127L is associated with genotypes E and F and HBsAg subtype determinant w4 and not w2. Only one mother (last case) had an HBV primary resistance mutation (M204V) against Lamivudine and Entecavir (upon prolonged Entecavir exposure).

### 3.3. Newborn Characteristics

**Figure 3:**
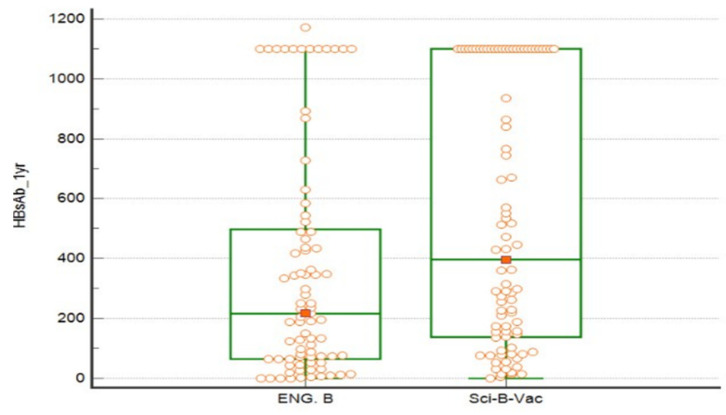
Shows the median and interquartile range (IQR) of newborn anti-HBs titers in the intention to treat analysis. Eng. B, Engerix-B. Wilcoxon rank-sum was used to compare the median values.

In the ITT analysis, the newborn gender distribution and neonatal gestational ages were similar between cohorts. The male: female rates in groups A and B were 61%:39% vs. 55.1%:44.9%, respectively, similarly, in the PPA there was no difference in newborn gender distribution, 61%:39% in group A vs. 53.1%:46.9% in group B, for male and female, respectively (*p* = 0.2). At one year post-delivery, a lower vaccine failure rate was observed in infants in group B (Sci-B-Vac group). More specifically, the proportion of infants who had non-protective anti-HBs titers <10 mIU/mL within one year of delivery was marginally lower among group B compared to group A (2.2% vs. 9.9%, *p* = 0.05, respectively in the ITT analysis and 2.5% vs. 9.9%, *p* = 0.09, respectively in the PPA) (Figure 3).

Moreover, a marginally higher proportion of infants in group B in the ITT analysis and PPA achieved higher anti-HBs titers of >10 mIU/mL (97.7% and 97.5%), compared to group A (90.1%), respectively, (OR 4.7, 95% CI 0.96–22.6, *p* = 0.05 and OR 4.3, 95% CI 0.9–21.1, *p* = 0.09), respectively (Table 3). However, the median ± IQR anti-HBs titers at one-year were significantly higher in group B (396.8 ± 962.1 mIU/mL), as compared to group A (216.1 ± 425.6 mIU/mL), *p* = 0.008 and 362.15 ± 957.3 mIU/mL in group B vs. 216.1 ± 425.6 mIU/mL in group A, *p* = 0.009 in the PPA. A statistically significant higher rate of infants reached very high levels of >1000 mIU/mL of 32.3% and 14.8% in the ITT (OR 2.7, 95% CI 2.7–5.84) and 33.3% and 14.8% in the PPA (OR 2.87, 95% CI 1.33–6.19, *p* = 0.009) for groups B and A, respectively. Overall, six newborns were HBsAg-positive at 12 months of age. In the Engerix-B group, one HBsAg positive infant had positive anti-HBs with a titer of 249.8 mIU/mL, while the rest of 5 HBV infected infants had negative anti-HBs. Notably, the use of Sci-B-Vac was associated with a lower incidence of HBsAg positivity as compared to Engerix-B (1.1% and 1.2 vs. 6.1%) in the ITT and PPA, respectively, (risk difference of −0.05, 95% CI −0.11–0.007, *t*-test = 0.05) (Table 3). Further analysis of infant HBV status at one year among mothers who were not treated by anti-viral treatments during pregnancy showed insignificant difference of HBsAg and anti-HBc positivity rate among the various maternal viral loads cut-off levels (Table 4).

Table 5 demonstrates the neonatal and the maternal characteristics of the six presumed MTCT cases. Three mothers of the 6 HBsAg+ infants had at delivery > 7 log_10_ IU/mL HBV DNA, one mother has 5.5 log_10_ and two mothers had <1.28 log_10_. Taken together, active/passive vaccination was effective in 10/13 cases with maternal HBV DNA levels > 7 log10 IU/mL up to 9.5 log10 IU/mL, but failed in 3 cases for unknown reasons. Notably, two cases in group A (case number 1 and number 5, Table 5) had an undetectable viral load ≤ 1.28 and HBeAg was negative. Irrespective of the source of infection, the active /passive vaccination at birth with Engerix-B should have protected the infants.

**Figure 4:**
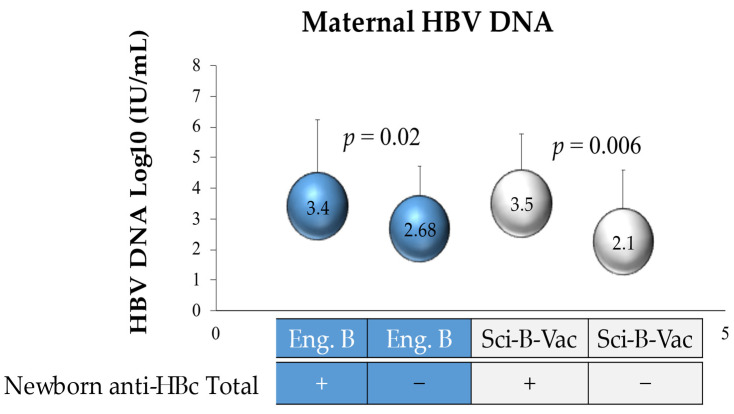
HBV DNA log 10 of mothers of anti-HBc-positive newborns. Eng. B, Engerix-B.

The active vaccine type had no significant impact on the rate of anti-HBc positivity at the one year follow up (Table 3). In the ITT analysis, the HBV DNA levels were measured in mothers of anti-HBc positive infants in groups A vs. B, demonstrating no difference (3.4 and 3.5 logs, respectively, *p* = 0.4). Likewise, similar mean maternal HBV DNA levels (*p* = 0.09) were measured in group A versus B with anti-HBc negative infants (2.68 and 2.1 logs, respectively) (Figure 4). However, mean maternal HBV DNA levels were significantly (*p* = 0.02 and 0.006) higher in the anti-HBc positive infants of groups A & B as compared to the anti-HBc negative infants (Figure 4).

## 4. Discussion

Mother to child HBV transmission (MTCT) is a significant route of HBV transmission, accounting for 40–50% of HBV infections in chronic carriers, particularly in highly endemic areas [14,15]. MTCT prevention by universal infant HBV vaccination has considerably reduced the global burden of HBV morbidity and mortality as it prevents acute infections in infants and children and the life-long consequences of chronic HBV infection, liver cirrhosis and hepatocellular carcinoma [16,17,18,19,20]. The benefits of screening pregnancies for HBsAg include identification not only of high-risk neonates who require immediate passive immune prophylaxis and a birth dose of the vaccine, but also of women who might need treatment, and of sexual partners and household members who could benefit from testing, counseling, vaccination, or therapy [15]. MTCT still can occur despite immune prophylaxis with HBIG passive immunization and active HBV vaccination. Maternal HBeAg positivity and high viral load are the most important risk factors for vaccine failure [21,22,23]. However, little is known about potentially different efficacies between various types of hepatitis B vaccines.

Our study demonstrated that the use of Sci-B-Vac might be more effective in preventing perinatal transmission of HBV mainly of genotype D even from mothers with high viral load, as compared to Engerix-B, as it was associated with significantly higher anti-HBs titers at one-year as compared to the Engerix-B coupled with a trend of significance for a lower HBsAg positivity rate.

These results are in line with previous studies. Use of Sci-B-Vac was associated with induction of more rapid and stronger anti-HBs responses in healthy children and newborns [24].

Several studies have implicated high maternal viremia as the most important factor associated with failure of neonatal vaccination. HBV DNA viral load of more than 1.1 × 10^7^ IU/mL correlated with a 32% HBV transmission rate compared to 0% transmission rate at HBV DNA viral loads of less than 1.1 × 10^7^ IU/mL [25,26]. Further studies have shown a linear relation between maternal viral DNA levels and the risk of immune-prophylaxis failure, which is reported in approximately 10–30% of cases [27,28,29]. Short-term antiviral therapy with drugs such as tenofovir for pregnant women with high HBV viral load during the third trimester has been recommended by international expert societies like the European Association for the Study of the Liver (EASL) [28], the American Association for the Study of Liver Diseases (AASLD) [29] to further decrease mother-to-infant HBV transmission in the post-vaccination era. Recently, the WHO reported as well that peripartum antiviral prophylaxis is highly effective at reducing the risk of HBV perinatal transmission, especially using tenofovir disoproxil fumarate [7,30]. In our study, there were 16 pregnant women with high viremic state with HBV DNA levels ≥ 7 log_10_ IU/m that were treated with NUC’s at mid-pregnancy; none of their babies had detectable HBsAg after one year suggesting the efficacy of HBV screening and antiviral treatment during pregnancy.

On the other hand, MTCT in this study occurred only in 3 cases (23%, 2/3 were Engerix-B failures) out of the untreated 13 women who had log_10_ > 7 at delivery. In one MTCT case the mother was HBeAg positive with moderately high viremia, log10 = 5.5 which is a level which may lead to MTCT. However, in two cases the HBV DNA assay was negative or very low with log_10_ ≤ 1.28 with negative HBeAg. This could suggest that a false negative result of the HBV DNA assay in these two cases cannot be excluded or the infant was infected soon after birth from another source because MTCT with undetectable virus loads are virtually unknown [31]. Due to restricted sample volume, we could not sequence the HBV DNA in the infected infants. Thus, we could not definitively prove that the HBV in the Engerix-B vaccinated infants came from the infected mother. However, irrespective of the timing or infection source, our study demonstrates more complete protection of the infants by Sci-B-Vac.

The significance of the anti-HBc found in approximately 30% of the infants one year after delivery is not clear. It is probably a remnant of the anti-HBc passively transferred from mother to the baby before delivery. The fact that mothers of anti-HBc positive infants had a slightly higher HBV DNA level could be understood as hint that HBV transmission occurred in some of the infants. But all mothers of HBsAg negative infants had HBV DNA levels < 100.000 IU/mL and were HBeAg negative. Thus, it is more probable that the slightly higher maternal HBV DNA level was associated with a higher maternal anti-HBc level which led to longer detectability of anti-HBc in the infant.

Engerix-B contains 10 µg and Sci-B-Vac 5 µg of total HBsAg; both vaccines contain the same adjuvant, use an identical vaccination regimen and both recombinant vaccines originate from HBV genotype A2. The genomic analysis in this study did not find any vaccine escape mutations which could explain the observed failures of Engerix-B. The maternal HBV genotyping demonstrated mostly subgenotype D1 in our cohort. Differences in the vaccine genotype and the infecting virus may reduce protection as observed in newly infected blood donor candidates of the US Red Cross [32] and may be an explanation for the variable failure rates of perinatal immune prophylaxis with conventional HBV vaccines as pointed elsewhere [33]. Our results suggest that Sci-B-Vac may improve the success rate of perinatal immune prophylaxis and this may possibly also apply for other heterologous maternal HBV genotypes than D1 which is prevailing in Middle East. Three properties of Sci-B-Vac may contribute to its potential superiority over the conventional vaccines like Engerix-B: (i) It is possible that the three-mammalian cell-derived HBV surface antigens in Sci-B-Vac contain only protection-inducing conformational HBsAg epitopes while HBsAg in Engerix-B from yeast cells is partially misfolded, which may result in antibodies against non-protective linear HBsAg epitopes [34]. (ii) The enhanced Sci-B-Vac efficacy may also be due to B-cell epitopes in the preS antigens that are absent in Engerix-B. The preS1 domain of HBV mediates virus attachment and entry to hepatocytes [35] and anti-preS1 and anti-preS2 antibodies were shown to neutralize HBV infectivity [36,37]. (iii) Furthermore, the HBV preS1 and preS2 domains contain highly effective T helper epitopes which may enhance the antibody response against the HBsAg, and may have additional protective effects [38] as suggested by the findings of this study.

The limitations of our study include that all pregnant women were recruited into the study at the delivery room, as this led to imbalance of treating women with high viremia during pregnancy with antivirals (6/82 in the Engerix-B vs 10/89 in the Sci-B-Vac groups), the second limitation is that the difference was marginally significant, and the last one is that we analyzed only small number of samples for genetic testing due to the fact that we could not obtain more peripheral blood samples from the other recruited mothers.

## 5. Conclusions

In conclusion, Sci-B-Vac might be more effective than Engerix-B in preventing HBV infection of genotype D in early childhood and may contribute to achieve the WHO goal for global elimination of HBV infection as a public health hazard. Further studies are needed to validate our findings.

## Figures and Tables

**Table 1:** Baseline maternal laboratory results at the delivery room.

	Intention to Treat Analysis			Per Protocol Analysis		
Variables	Group A (*n* = 82)	Group B (*n* = 89)	*p* Value	Group A (*n* = 82)	Group B (*n* = 81)	*p* Value
	Engerix-B	Sci-B-Vac		Engerix-B	Sci-B-Vac	
Age, (median ± IQR)	27.03 ± 9.53	28 ± 8.02	0.15	27.03 ± 9.53	28.3 ± 9.14	0.09
ALT (U\L), (median ± IQR)	19 ± 11	16 ± 14	0.3	19 ± 11	16 ± 15	0.3
AST (U\L), (median ± IQR)	22 ±12	21 ± 13	0.7	22 ± 12	21 ± 13.5	0.8
WBC (thousands), (median ± IQR)	6.86 ± 2.8	7.2 ± 2.42	0.4	6.86 ± 2.8	7.4 ± 2.43	0.3
Hb (g\dl), (median ± IQR)	12.2 ± 1.6	11.7 ± 2	0.054	12.2 ± 1.6	11.7 ± 2	0.051
PLT (thousands), (median ± IQR)	230 ± 62	200 ± 82	0.051	230 ± 62	201 ± 90	0.06
INR, (median ± IQR)	1.04 ± 0.12	1.01 ± 0.1	0.02	1.04 ± 0.12	1.01 ± 0.11	0.03
HBsAg+ N (%)	82 (100)	89 (100)	-	82 (100)	81 (100)	-
HBeAg+ N (%)	7 (8.54)	10 (11.24)	0.44	7 (8.54)	9 (11.1)	0.4
Anti-HBe+ N (%)	74 (90.24)	69 (77.53)	0.58	74 (90.24)	64 (79.01)	0.7
**HBV DNA status**						
Positive mothers, N	67 (81.7)	69 (77.5)	0.1	67 (81.7)	63 (77.8)	0.2
>log_10_ IU/mL, (median ± IQR)	2.6 ± 2.14	2.1 ± 3.36	0.19	2.6 ± 2.14	2.33 ± 3.53	0.2
* Maternal viral loads, N (%)						
Undetectable viral load	14 (17.1)	20 (22.5)	0.4	14 (17.1)	19 (23.5)	0.3
Detectable–1999 IU/mL	39 (47.6)	40 (44.9)	0.9	39 (47.6)	37 (45.7)	0.9
2000–199,999 IU/mL	16 (19.5)	14 (15.7)	0.6	16 (19.5)	11 (13.6)	0.3
200,000–7 log_10_	5 (6.1)	4 (4.5)	0.8	5 (6.1)	4 (4.9)	0.8
>7 log_10_	7 (8.5)	7 (7.9)	0.7	7 (8.5)	6 (7.4)	0.8
** Maternal viral loads, N (%)						
Undetectable viral load	14 (17.3)	20 (23.5)	0.4	14 (17.3)	19 (24.7)	0.3
Detectable–1999 IU/mL	39 (48.1)	40 (47.1)	0.9	39 (48.1)	37 (48.1)	0.9
2000–199,999 IU/mL	16 (19.8)	14 (16.5)	0.6	16 (19.8)	11 (14.3)	0.3
200,000–7 log_10_	5 (6.2)	5 (5.9)	0.8	5 (6.2)	4 (5.2)	0.8
>7 log_10_	7 (8.6)	6 (7.1)	0.7	7 (8.6)	6 (7.8)	0.8
**Anti-viral treatment, N (%)**						
Lamivudine	2 (2.4)	2 (2.5)	0.2	2 (2.4)	2 (2.5)	0.2
Telbivudine	4 (4.8)	4 (4.9)	0.2	4 (4.8)	4 (4.9)	0.2
Tenofovir	0	4 (4.9)	0.1	0	4 (4.9)	0.1

* Analysis including the five mothers with missing viral loads (1 in group A and 4 in group B) in the per protocol analysis (PPA) and the intention to treat (ITT) analysis. ** Analysis excluding the five mothers with missing viral loads (1 in group A and 4 in group B) in the PPA and the ITT analysis.

**Table 2:** HBV subgenotype, HBsAg subtype and S gene mutations in selected 9 cases with positive anti-HBc in the infant at month 12.

Serial Number of Mothers Who Had Genetic Analysis									
	1	2	3	4	5	6	7	8	9
HBV subgenotype	D1	D1	D2	D1	D1	D1	D1	D1	D2
HBsAg subtype	ayw2	ayw2 or 4	ayw3	ayw2	ayw2	ayw2	ayw2	ayw2	ayw2
Mutations in RT	I266T	-	M129L, E271D	R110F	R110G, S119P, L164M Q215S	N238D	L157M, V214A, Q215S, I266K	F221Y	**M204V,** P237H
Mutations in S gene	P203R	**T127L**	T118A	S174N	Y200F, Y206F, S207R	-	S193L, Y206H, S207R	S207N, L213I	-
Vaccine immune escape	no	no	no	no	no	no	no	no	no
Drug resistance	no	no	no	no	no	no	no	no	LMV, (ETV)
**Maternal Characteristics**									
Viral load IU/mL	1860	<15	319000	83.9	16000	10093	<15	109	150
HBeAg	Negative	Negative	Positive	Negative	Negative	Negative	Negative	Not available	Negative
Therapy	None	None	None	None	None	None	None	None	Entecavir
ALT (U/L)	9	20	14	16	18	15	342	-	18
**Infants’ Characteristics**									
HBsAg	Negative	Negative	Positive	Negative	Negative	Negative	Negative	Negative	Negative
Anti-HBs titer mIU/mL	123.4	1100	5.68	1100	1100	174.9	211.1	1100	76.9
Anti-HBc	Positive	Positive	Positive	Positive	Positive	Positive	Positive	Positive	Positive

**Table 3:** Neonatal characteristic in regard to HBV status.

	Intention to Treat Analysis			Per Protocol Analysis		
Variables, N (%)	Group A (*n* = 82)	Group B (*n* = 89)	*p* value	Group A (*n* = 82)	Group B (*n* = 81)	*p* value
	Engerix-B	Sci-B-Vac		Engerix-B	Sci-B-Vac	
HBsAg+	5 (6.1)	1 (1.1)	0.05	5 (6.1)	1 (1.2)	0.05
Anti-HBs titers						
<10 mIU/mL	8 (9.8)	2 (2.2)	0.05	8 (9.9)	2 (2.5)	0.09
>10 mIU/mL	73 (90.1)	85 (97.7)	0.05	73 (90.1)	79 (97.5)	0.09
>100 mIU/mL	52 (64.2)	68 (78.2)	0.05	52 (64.2)	64 (79)	0.05
>1000 mIU/mL	12 (14.8)	28 (32.2)	0.01	12 (14.8)	27 (33.3)	0.009
Anti-HBc positivity	28 (34.1)	26 (29)	0.4	28 (34.1)	23 (28.4)	0.4
Anti-HBc positivity according to maternal viral loads at delivery						
Undetectable viral load	6 (7.3)	1 (1.1)	0.04	6 (7.3)	1 (1.2)	0.055
Detectable–1999 IU/mL	11 (13.4)	13 (14.6)	0.8	11 (13.4)	13 (16)	0.6
2000–199,999 IU/mL	5 (6.1)	5 (5.6)	0.9	5 (6.1)	4 (4.9)	0.7
200,000–7 log_10_	2 (2.4)	2 (2.2)	0.9	2 (2.4)	2 (2.5)	0.99
>7 log_10_	4 (4.9)	3 (3.4)	0.6	4 (4.9)	3 (3.7)	0.7

**Table 4:** Fetal HBsAg and anti-HBc status among mothers who were not treated by antivirals during pregnancy.

	**Intention to Treat Analysis**			**Per Protocol Analysis**		
**Variables, N (%)**	**Group A (*n* = 75)** **Engerix-B**	**Group B (*n* = 75)** **Sci-B-Vac**	***p* Value**	**Group A (*n* = 75)** **Engerix-B**	**Group B (*n* = 67)** **Sci-B-Vac**	***p* Value**
HBsAg positivity in children according to Maternal viral loads						
Undetectable viral load	1/11	0/18	0.3	1/11	0/17	0.3
	(9.1%)	0		(9.1%)	0	
Detectable–1999 IU/mL	1/39	0/37	1	1/39	0/34	1
	(2.6%)	0		(2.6%)	0	
2000–199,999 IU/mL	0/14	0/14	1	0/14	0/11	1
	0	0		0	0	
200,000–7 log_10_	1/4	0/3	1	1/4	0/2	1
	(25%)	0		(25%)	0	
>7 log_10_	2/7	1/3	0.9	2/7	1/3	0.6
	(28.6%)	(33.3%)		(28.6%)	(33.3%)	
Anti-HBc positivity in children according to Maternal viral loads						
						
Undetectable viral load	3/11	1/18	0.09	3/11	1/17	0.1
	(27.3%)	(5.6%)		(27.3%)	(5.9%)	
Detectable–1999 IU/mL	11/39	12/37	0.6	11/39	12/34	0.5
	(28.2%)	(32.4%)		(28.2%)	(35.3%)	
2000–199,999 IU/mL	5/14	5/14	1	5/14	4/11	0.9
	(35.7%)	(35.7%)		(35.7%)	(36.4%)	
200,000–7 log_10_	1/4	2/3	0.3	1/4	2/2	0.4
	(25%)	(66.7%)		(25%)	(100%)	
>7 log_10_	5/7	3/3	0.9	5/7	3/3	0.9
	(71.4%)	(100%)		(71.4%)	(100%)	

Analysis excluding the six treated mothers with antivirals in group A and 10 mothers in group B, and excluding the five mothers with missing viral loads (1 in group A and 4 in group B) in the PPA and the ITT analysis.

**Table 5:** Characteristics of the presumed six mother to child HBV transmission (MTCT) cases.

Infant Characteristics at 12 Months					
Case Number	Gender	Administered Vaccine	HBsAg	Anti-HBs Titer mIU/mL	Anti-HBc
1	Male	Engerix-B	Positive	249.8	Positive
2	Male	Engerix-B	Positive	5.68	Positive
3	Male	Engerix-B	Positive	3.16	Positive
4	Female	Engerix-B	Positive	0.85	Positive
5	Female	Engerix-B	Positive	0.2	Positive
6	Female	Sci-B-Vac	Positive	0.4	Positive
**Maternal Characteristics**					
**Case number**	**Age at delivery (Years)**	**Antiviral therapy**	**HBeAg**	**Anti-HBe**	**HBV DNA Log at Delivery**
1	23	No	Negative	Positive	<1.28
2	25	No	Positive	Negative	5.5
3	32	No	Positive	Negative	8.49
4	21	No	Negative	Positive	9.53
5	19	No	Negative	Positive	<1.28
6	26	No	Positive	Negative	9.00

## Data Availability

The data are available in the Liver unit at Hadassah Medical Organization and will be available upon reasonable request

## Abbreviations

HBV	hepatitis B virus
HBsAg+	hepatitis B surface antigen positive
HBIG	Hepatitis B Immune Globulin
INR	international normalized ratio
HBsAg	hepatitis B surface antigen
HBeAg	hepatitis B e antigen
anti-HBe	antibody to hepatitis B e antigen
HCV	hepatitis C virus
HDV	hepatitis D virus
anti-HBc	antibody to hepatitis B core antigen
anti-HBs	antibody to hepatitis B surface antigen
WHO	World Health Organization
MTCT	mother to child HBV transmission
IM	intramuscular
ALT	alanine aminotransferase
AST	aspartate transferase
PCR	polymerase chain reaction
SD	standard deviation
NUC	nucleoside analogues
EASL	Association for the Study of the Liver
AASLD	American Association for the Study of Liver Diseases
